# Rapid Genetic Divergence of an Invasive Species, *Spartina alterniflora*, in China

**DOI:** 10.3389/fgene.2020.00284

**Published:** 2020-04-24

**Authors:** Lu Xia, Qifang Geng, Shuqing An

**Affiliations:** ^1^School of Life Sciences, Nanjing University, Nanjing, Jiangsu, China; ^2^Nanjing University Ecology Research Institute of Changshu (NJUecoRICH), Changshu, China; ^3^Asian Natural Environmental Science Center, The University of Tokyo, Bunkyo, Japan

**Keywords:** gene flow, genetic diversity, genetic structure, hybridization, multiple introductions, rapid evolution

## Abstract

Hundreds of plants and half a kilogram of seeds of *Spartina alterniflora*, which were collected from Morehead City in North Carolina, Sapelo Island in Georgia, and Tampa Bay in Florida, were introduced to China in 1979. However, according to documented records, *S. alterniflora* from different origins were introduced to different areas when the species was first introduced to the coastal areas of China in the 1980s. In order to understand the relationship between the invasive *S. alterniflora* populations of China and the native *S. alterniflora* populations of the United States, and whether the genetic structure and genetic diversity of the invasive populations of China were affected by different introductions in the 1980s, molecular markers were used to determine the levels of gene flow and its effect on population differentiation. A total of 715 samples of *S. alterniflora* were collected from nine invasive populations in China and nine native populations from the United States. The genetic diversity and genetic structure of invasive and native populations were compared using microsatellite markers. The heterozygosity of Chinese invasive populations of *S. alterniflora* (*H_O_* = 0.538, *H_E_* = 0.725) were similar with those of native populations (*H_O_* = 0.530, *H_E_* = 0.744), which may attribute to its multiple introductions with the multisource populations from different geographic areas of the United States. However, the lower allelic diversities of Chinese invasive populations were detected, which may be due to the founder effect, or the bottleneck, which supports the theory that the allelic diversity is more sensitive to population bottlenecks than heterozygosity. The results of the STRUCTURE analysis among all sampling sites showed that the value of ΔK was largest when *K* = 2, which indicated that the invasive *S. alterniflora* populations in China had completed differentiated from the native populations of the United States. This may be because of admixture and hybridization of three non-overlapping original populations, or the postintroduction rapid evolution in China, and reproductive isolation under long-term geographic isolation. There was significant differentiation among invasive populations, which was mainly affected by different human-mediated introductions in 1980s. Significant genetic structure (*K* = 7) and high genetic differentiation (*Fst* = 0.30193) were detected in Chinese invasive populations, which may due to the low natural gene flow among populations. The genetic structure of the invasive populations in China was still affected by the human-mediated introductions in the 1980s, and the different initial introductions might promote differentiation among the invasive populations. In fact, the human-mediated long-distance dispersal should take the most of responsibility for the rapid spread of *S. alterniflora* along the coast of China. Multisource introductions of *S. alterniflora* are perhaps helpful for local adaptation but itself cannot cause rapid spread along the whole coast of China. Meanwhile, we suggest that the prevention of gene exchange among populations of *S. alterniflora* is the first and most important step in the control of the species on the coast of China, because admixture and hybridization of isolated populations might generate new heterosis and increase the difficulty of managing *S. alterniflora* in China.

## Introduction

The genotype, genetic diversity, and genetic structure of an invasive plant species always differ from those of native congeners. This may be due to rapid evolution after invasion, outcrossing, or genotypes with specific traits being better able to invade (Lee, 2002; Parker et al., 2003). Individuals colonizing new ranges likely face environments different from those previously experienced (van Boheemen et al., 2019). Nonetheless, rapid evolutionary changes have been recently found to be widespread in invasive species and have been proposed as a precursor to successful invasions (Xu et al., 2015). Some successful invasions are the product of hybridization, either between genetically distinct allopatric from the same species or between closely related but otherwise allopatric species, brought together by introductions into new locations (Reznick et al., 2019). Heterosis, or hybrid vigor, refers to the phenomenon that progeny of diverse inbred varieties exhibits greater biomass (Birchler et al., 2003), tolerance (Gallego-Tevar et al., 2018), and so on, than the better of the two parents.

In addition, when invasive species are establishing in the new range, they often suffer founder effects, bottlenecks, and eventually genetic drift as a result of finite number of individuals in the new colony, which can considerably decrease the genetic variation and change the allelic frequencies compared to the populations in the regions of origin (Guo et al., 2018). On the other hand, gene flow, either via multiple introductions from the original range, propagule dispersal (gametes/individuals) in the new range, outcrossing, and novel genetic admixtures, can mitigate founder effects by increasing genetic diversity and facilitate adaptation in the new range (Guo et al., 2018).

However, landscape factors and environmental conditions have a strong influence on gene flow and shape the spatial genetic variation accordingly. Both isolation by distance (Jenkins et al., 2010; Guo et al., 2018) and isolation by environment (Wang and Bradburd, 2014; Guo et al., 2018) have been proposed to explain the spatial variation patterns of plants. Isolation by distance and isolation by environment, as the geographic distance and environmental distance are often correlated and both play an important role in introduced species (Shafer and Wolf, 2013; Wang, 2013; Guo et al., 2018). Meanwhile, anthropogenic activities also contribute to the spatial distribution of genetic diversity. Anthropogenic activities provide dispersal corridors for invasive species and facilitate gene exchange within the introduced range, or even between the native and introduced ranges (e.g., by multiple introductions), and can thus mitigate the effects of isolation by distance and isolation by environment (Guo et al., 2018).

Marine invasions represent a global threat to human populations and broader biological communities and are often listed as one of the top conservation concerns worldwide (Blakeslee et al., 2019). *Spartina alterniflora* is a perennial herb that is native to the lower intertidal salt marshes along the Atlantic and Gulf coasts of North America (Wang et al., 2006). In 1979, hundreds of plants and half a kilogram of seeds of *S. alterniflora* were introduced in China for ecological engineering from populations in Morehead City in North Carolina, Sapelo Island in Georgia, and Tampa Bay in Florida by Zhong and his colleagues of Nanjing University (Xu and Zhuo, 1985). Because of different introductions over a large geographic area, *S. alterniflora* rapidly spread across the coastal marshes of China, with its current range now extending from Liaoning in north to Guangxi in the south (Xia et al., 2015; Qiao et al., 2019). In fact, there have been multiple introductions of *S. alterniflora* into Willapa Bay, USA, and new genotypes have been generated by admixture following secondary contact among previously allopatric native populations (Civille et al., 2005). Because the origins of China’s *S. alterniflora* are three allopatric native populations in the United States, An et al. (2007) and Xia et al. (2015) considered that admixture and hybridization of *S. alterniflora* of different origins might increase genetic variation within the invasive population, and hybrids have stronger environmental tolerance and thus have higher growth rates than native congeners. Because of heterosis, the offspring produced by intraspecific hybridization often show stronger adaptability than their parents; therefore, multiple introductions were considered an important factor to promote successful invasion (Geller et al., 2010). Qiao et al. (2019) found “hybrid swarms” in coastal areas of China, and the invasion ability of “hybrid swarms” was far greater than that of the original *S. alterniflora*.

However, all seedlings and seeds collected from the United Stated were planted separately, in Luoyuanwan in Fujian Province in 1981 (Qin et al., 1985), according to their origins, and then *S. alterniflora* of different origins were gradually introduced into different areas on the coast of China (Xu and Zhuo, 1985), and according to documented records, *S. alterniflora* was not uniformly introduced from Luoyuanwan to other coastal areas of China (Xu and Zhuo, 1985; Xia et al., 2015; Table 1).

**TABLE 1:** Sources of Spartina alterniflora in each population on the coast of China when it was introduced in the 1980s.

Province/City	Number	Source	Time	References
Hebei (Tianjin)	101,100	North Carolina	1986	Xia et al., 2015; Zhao et al., 2015
Hebei (Tangshan)		Unknown	1980s	Zhao et al., 2015
Hebei (Cangzhou)		Unknown	1998	Zhao et al., 2015
Shandong	101,100; 301,300	North Carolina and Georgia	1983	Xu and Zhuo, 1985; Deng et al., 2006; Xia et al., 2015
Jiangsu (the abandoned Yellow River estuary)		Unknown	1982	Xia et al., 2015; Liu, 2018
Jiangsu (Yancheng)	301,300	Georgia	1983	
Jiangsu (Qidong)	301,300	Georgia	1983	
Jiangsu (Dongtai)		Unknown	1987–1988	
Zhejiang	201,200; 301,300	Georgia and Florida	1983	Lu and Zhang, 2013; Xia et al., 2015
Fujian	101,100; 201,200; 301,300	North Carolina, Georgia, and Florida	1981	Xu and Zhuo, 1985; Xia et al., 2015
Guangdong	201,200	Florida	1983	Xu and Zhuo, 1985; Deng et al., 2006; Xia et al., 2015
Guangxi	101,100; 201,200; 301,300	North Carolina, Georgia, and Florida	1986	Mo et al., 2010; Xia et al., 2015

**Number is numbered according to their original regions (101 or 100 for North Carolina, 201 or 200 for Florida, and 301 or 300 for Georgia, respectively, from seed-germinated and ramet-generated individuals).**

Direct associations between neutral marker variation and adaptive changes have been experimentally found (Santos et al., 2012; Vilas et al., 2015), and neutral genetic diversity is helpful for assessing the evolutionary potential of invasive species (Xu et al., 2015). Microsatellites are one of the most popular neutral markers because of their abundance, high polymorphism, Mendelian codominant inheritance, and rapid and convenient detection (Ellegren, 2004; Selkoe and Toonen, 2006). In addition, the comparison of genes between native and invasive *S. alterniflora* populations is of great significance for the study of the postintroduction rapid evolution and increasing genetic diversity in admixture and intraspecies hybridization involving secondary contact after invasion (Blum et al., 2007). In particular, the human-mediated introductions have provided unplanned experiments to help us better understand whether multisource introductions increase the genetic diversity within invasive populations (Sax et al., 2007) and how anthropogenic factors affect gene flow patterns and the spatial genetic patterns underpinning the expansion and distribution of invasive species. In order to test our hypotheses, in this study, the genetic diversity and structure of the invasive populations and the native populations were compared using microsatellite markers. We also want to understand the relationship between the invasive *S. alterniflora* populations of China and the native *S. alterniflora* populations of the United States and whether the genetic structure and genetic diversity of the invasive populations of China have been affected by different introductions from different sources during the 1980s. Molecular markers were used to estimate the level of gene flow and determine its effect on population differentiation. Based on the results, we give some suggestions for the control of *S. alterniflora* along the coast of China.

## Methods and Data Analyses

### Documented Records of Introductions

**FIGURE 1:**
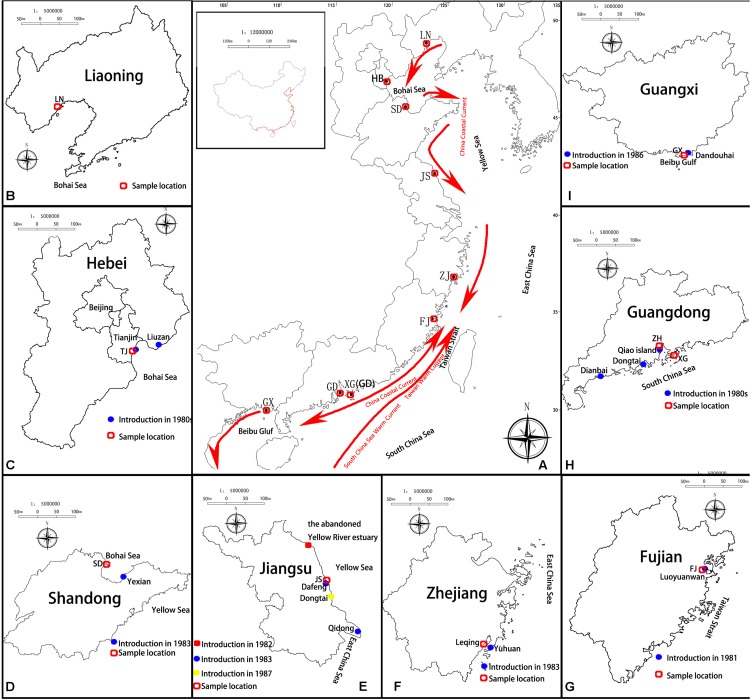
Introduction and sampling sites of *Spartina alterniflora* on the coast of China. **(A)** General view; the red arrow shows the direction of the China coastal current, and the introduction sites of **(B)** Liaoning Province; **(C)** Hebei Province (Tianjin); **(D)** Shandong Province; **(E)** Jiangsu Province; **(F)** Zhejiang Province; **(G)** Fujian Province; **(H)** Guangdong Province; and **(I)** Guangxi Province.

According to documented records, all the living individuals and the germinated seedlings of *S. alterniflora* collected from America were planted in the nurseries in Luoyuanwan, Fujian Province, in 1981 (Qin et al., 1985; Xu and Zhuo, 1985). These nursery populations formed the founder population of *S. alterniflora* in China. The nursery area was 1,000–1,500 m^2^, and each individual was planted in rows 0.5 m apart with 0.5 m between individuals, separating *S. alterniflora* of different origins (Qin et al., 1985). Later, the seeds produced from the nursery plantations were collected, numbered according to their original regions (101 or 100 for North Carolina, 201 or 200 for Florida, and 301 and 300 for Georgia, respectively, from seed-germinated and ramet-generated individuals), and stored from 1982 to 1985 (Xu and Zhuo, 1985; Xia et al., 2015). The seeds and seedlings that were produced by the founder populations of *S. alterniflora* were the first introductions of *S. alterniflora* to the coastal areas of China. Most Chinese researchers at that time considered that *S. alterniflora* of North Carolina was the most cold-resistant, *S. alterniflora* of Florida was the most heat-resistant, and *S. alterniflora* of Georgia was the most suitable for middle latitudes (Qin et al., 1985; Chen and Chung, 1990). Thus the offspring of *S. alterniflora* from North Carolina were introduced to northern China, the offspring of *S. alterniflora* from Georgia were introduced to the middle latitudes of China, and the offspring of *S. alterniflora* from Florida were introduced to southern China (Qin et al., 1985; Chen and Chung, 1990; Xia et al., 2015). We list each introduction in chronological order. In March 1981, the second trial planting was conducted in Luoyuanwan, Fujian (FJ; no. 101 and 100, no. 201 and 200, no. 301 and 300; Xia et al., 2015; Lu and Wang, 2017). 1n 1983, trial plantings were conducted in Dafeng District, Yancheng, on the beach of Qidong, Jiangsu Province (JS; no. 301 and 300; Xia et al., 2015; Liu, 2018), on the beach of Yuhuan, Zhejiang Province (ZJ; no. 201 and 200, no. 301 and 300; Xia et al., 2015; Lu and Wang, 2017), and on Aoqi Island, Zhuhai and in Baidian District, Maoming, Guangdong Province (GD; no. 201 and 200; Xia et al., 2015; Lu and Wang, 2017). From 1985 to 1986, trial plantings were conducted on the beach of Tianjin (TJ; no. 101 and 100; Xia et al., 2015; Zhao et al., 2015), on the beach of Yexian, Shandong Province (SD; no. 101 and 100; no. 301 and 300; Xu and Zhuo, 1985; Deng et al., 2006), and in Dandouhai, Beihai, Guangxi Province (GX; no. 101 and 100, no. 201 and 200, no. 301 and 300; Xia et al., 2015; Lu and Wang, 2017; Table 1 and Figure 1). In addition, in 1982, a trial planting was conducted in the abandoned Yellow River estuary, Jiangsu Province, without knowing the origin of the planted *S. alterniflora* individuals (Table 1). In 1987 or 1988, a trial planting was conducted in Dongtai, Jiangsu Province, without knowing the origin of the *S. alterniflora* individuals (Xia et al., 2015; Zhao et al., 2015; Liu, 2018; Table 1). In the 1980s, a trial planting was conducted in Liuzan Town, Tangshan, Hebei Province, which neighbors Tianjin, without knowing the origin of the *S. alterniflora* individuals (Zhao et al., 2015; Liu, 2018; Table 1). From 1981 to 1985, the exact year was not clear, another batch of seedlings of *S. alterniflora* was introduced from Florida to Taishan County, Guangdong Province (Deng et al., 2006; Table 1).

### Study Sites and Sampling

A total of 371 individuals of *S. alterniflora* were collected from nine non-native populations throughout its entire distribution range in China: Liaoning (LN), Hebei (HB), Shandong (SD), Jiangsu (JS), Zhejiang (ZJ), Fujian (FJ), Guangdong (GD), Hongkong (XG), and Guangxi (GX) (Figure 1). In addition, a total of 344 individuals of *S. alterniflora* were collected from nine populations in three states of the United States: North Carolina (NC), Georgia (GA), and Florida (FL) (Table 2). The sampling sites in China were located at or close to the sites of introductions, which were recorded in the literature (Table 1 and Figure 1), and we also collected samples from two sites where the species was not introduced but later spread to LN and XG. The sampling sites in the United States were located at or close to the well-documented source locations for *S. alterniflora* in China, and we chose three populations in each of the three states (Xu and Zhuo, 1985). To minimize the possibility of resampling clones, approximately 40 individuals were sampled from each location with a distance of 30 m between samples. For each leaf sample, 5–10 leaf pieces (<3 cm in length) were cut and put into a plastic Ziploc bag with silica gel for drying immediately, and then they were stored at room temperature until DNA extraction.

**TABLE 2:** Geographic location and population genetic parameters for each site sampled.

Code	Location	Latitude (N)	Longitude (E)	N	A	*A*_*R*_	*H*_*O*_	*H*_*E*_	*F*_*IS*_
LN	Huludao, Liaoning Province, China	40°52’42.48″	121°01’19.38″	33	2.125	2.014	0.270	0.224	−0.191
HB	Tianjin, China	38°59’06.00″	117°42’53.04″	32	6.000	5.605	0.606	0.674	0.118
SD	Dongying, Shandong Province, China	37°50’28.50″	119°05’15.54″	42	2.250	2.094	0.654	0.440	−0.478
JS	Yancheng, Jiangsu Province, China	33°16’07.44″	120°47’23.94″	38	4.875	4.627	0.500	0.651	0.245
ZJ	Wenzhou, Zhejiang Province, China	28°06’10.44″	121°00’14.58″	39	4.375	3.948	0.430	0.531	0.203
FJ	Putian, Fujian Province, China	25°15’14.10″	119°00’08.88″	22	5.375	5.335	0.633	0.627	0.015
XG	Xianggang, China	22°26’23.34″	113°56’53.58″	45	3.125	3.002	0.343	0.384	0.116
GD	Zhuhai, Guangdong Province, China	22°15’18.66″	113°35’20.58″	37	2.375	2.190	0.885	0.452	−0.957
GX	Beihai, Guangxi Province, China	21°41’07.20″	109°09’23.22″	56	6.375	5.730	0.555	0.665	0.174
NC3	Morehead City, NC, USA	34°43’18.69″	76°40’28.76″	40	7.375	6.630	0.534	0.625	0.158
NC2	Morehead City, NC, USA	34°42’11.98″	76°47’58.14″	40	7.500	6.897	0.553	0.634	0.146
NC1	Morehead City, NC, USA	34°42’01.21″	76°49’50.49″	41	7.750	6.898	0.566	0.672	0.170
GA3	Altamaha Estuary, GA, USA	31°27’17.35″	81°21’50.52″	42	8.125	7.181	0.524	0.662	0.222
GA2	Altamaha Estuary, GA, USA	31°25’11.72″	81°17’23.51″	43	9.125	7.855	0.518	0.725	0.296
GA1	Altamaha Estuary, GA, USA	31°24’54.84″	81°17’47.43″	41	8.375	7.414	0.538	0.700	0.245
FL3	Tampa Bay, FL, USA	29°51’06.25″	83°37’02.98″	41	7.250	6.404	0.484	0.612	0.221
FL2	Tampa Bay, FL, USA	29°51’01.44″	83°37’13.76″	42	7.625	6.548	0.518	0.640	0.202
FL1	Tampa Bay, FL, USA	29°50’34.85″	83°37’01.95″	41	8.500	7.120	0.530	0.683	0.237

**N, the number of analyzed samples; A, mean number of alleles per locus; AR, allelic richness, the mean allele number for eight microsatellite loci by using FSTAT; HO and HE, mean observed and expected heterozygotes for eight loci, respectively; FIS, mean inbreeding coefficients for eight loci; PA, the number of private alleles for all loci. A, HO, HE, and FIS were calculated using GENALEX.**

### DNA Extraction and Microsatellite Analysis

Total genomic DNA was extracted from each of the silica gel–dried leaf samples (<10 mg) using a modified cetyltrimethylammonium bromide method (Doyle and Doyle, 1987).

To investigate the characteristics of 35 microsatellite (SSR) loci isolated from *S. alterniflora* (Blum et al., 2004; Sloop et al., 2005), we randomly selected eight samples from eight Chinese populations. Of 35 SSR loci, eight (SPAR. 07, SPAR. 10, SPAR. 14, SPAR. 15, SPAR. 16, SPAR. 26, SPAR. 29, SPAR. 34) were codominant and highly polymorphic (Supplementary Table S1).

To amplify the SSR loci of *S. alterniflora*, we used a tailed primer method to perform polymerase chain reaction (PCR); that is, 5′ end of each forward primer was tailed with an M13 sequence (5′-GTTTTCCCAGTCACGAC-3′). Polymerase chain reaction amplification was carried out with a DNeasy Plant Mini Kit (Qiagen, Dússeldorf, North Rhine-Westphalia, Germany) in 30 μL reaction containing ≤100 ng of template genomic DNA, 1X GoTaq Colorless Master Mix and 0.2 μM of each of the three primers: a forward primer with a tail of the M13 sequence, a non-tailed reverse primer, and a 5′-labeled M13 primer (FAM, HEX, or TAMRA; General Biosystems, Chuzhou, Anhui, China).

Polymerase chain reaction cycling conditions were 5 min at 95°C, followed by 35 cycles of 15 s at 95°C, 30 s at 55°C, and 30 s at 72°C, with an extension of 10 min at 72°C in the final cycle. Polymerase chain reaction products were identified and analyzed with PTC200 (MJ Research, Bruno, QC, Canada).

### Data Analysis

#### Genetic Diversity

The mean number of alleles per locus (*A*), allelic richness (*A*_R_), the number of private alleles (P_A_), observed and expected heterozygosity (P_A_), and observed and expected heterozygosity (*H*_O_ and *H*_E_), at each of the populations were estimated using GENALEX version 6.4 (Peakall and Smouse, 2006). Inbreeding Inbreeding coefficients (*F*_IS_) were calculated using FSTAT version 2.9.3.2 (Goudet, 1995). In order to compare genetic diversities between the invasive and the native region, all the Chinese sampling sites were combined to form the Chinese invasive group, and all the American sampling sites were combined to form the American native group. The mean number of alleles per locus (*A*), allelic richness (*A*_R_), and observed and expected heterozygosity (*H*_O_ and *H*_E_) of the Chinese invasive group and the American native group were estimated and compared using GENALEX version 6.4 (Peakall and Smouse, 2006). According to the introduction records, in the 1980s, *S. alterniflora* introduced to each of the seven locations in China might be of different origins. *S. alterniflora* from a single-source were introduced to GD, *S. alterniflora* from two sources were introduced to SD and ZJ, *S. alterniflora* from multiple sources were introduced to FJ and GX, and *S. alterniflora* were introduced to HB and JS repeatedly (Table 1 and Figure 1). There were no initial introductions to LN and XG; therefore, we classified LN and XG in the non-source introduction group, GD in the single-source introduction group, SD and ZJ in the double-source introduction group, and HB, JS, FJ, and GX in the multisource/multi-introduction group. Analysis of variance was used to perform significance tests for the two parameters *A*_R_ and *H*_E_ against the latitudes and the number of sources of the nine populations in China (IBM SPSS Statistics for Windows, Version 22.0., IBM Corp, Armonk, NY, United States). We first excluded the populations with multiple introductions (i.e., HB and JS), and other populations with the same number of introduced sources were arranged from north to south. Then, we combined multiple introductions with multiple-source populations, and the populations in the same group were still listed from north to south.

#### Genetic Differentiation Among Populations

The collecting sites in each state of the United States were combined to one population for each state: NC_1_, NC_2_, and NC_3_ were combined to NC; GA_1_, GA_2_, and GA_3_ were combined to GA; and FL_1_, FL_2_, and FL_3_ were combined to FL (Table 3). ARLEQUIN version 3.5 (Excoffier and Lischer, 2010) was performed to estimate the total percentage variation attributable to genetic differentiation within and among populations of *S. alterniflora* in China and America. The genetic differentiation index between populations, pairwise *F*_ST_, was calculated using ARLEQUIN version 3.5 (Excoffier and Lischer, 2010), using 10,000 permutations. The significances of correlations between the genetic differentiation index (*F*_ST_) and geographic distance (in kilometers) of invasive and native populations were assessed using the Mantel test (Mantel, 1967) with 9,999 random permutations using GENALEX 6.5 software (Peakall and Smouse, 2006, 2012).

**TABLE 3:** Population genetic parameters of Chinese and American Spartina alterniflora.

	N	A	*A*_*R*_	*H*_*O*_	*H*_*E*_
China	344	4.097	3.838	0.542	0.516
USA	371	7.958	6.994	0.529	0.611

**N, the number of analyzed samples; A, average value of the number of alleles among Chinese or American populations of S. alterniflora; AR, allelic richness, average allele number among Chinese or American populations of S. alterniflora; HO and HE, average value of observed and expected heterozygotes among Chinese or American populations of S. alterniflora, respectively.**

#### Population Genetic Structure

Three methods were used to examine population genetic structure. First, we estimated patterns of genetic differentiation derived from pairwise *F*_ST_ values between Chinese and American populations, and then we estimated pairwise *F*_ST_ values among three populations (NC, GA, and FL) within America with population assignments using GENALEX version 6.4 (Peakall and Smouse, 2006). Biplots of pairwise population assignment likelihood values provided reliable graphical information about statistical power for population assignment (Peakall and Smouse, 2006). Second, the genetic distance matrix (Nei, 1972) obtained from Yeh et al. (1999) was used to perform a cluster analysis using the unweighted pair-group method with arithmetic average (UPGMA) using MEGA 5.0 (Tamura et al., 2011). Third, the population genetic structure was inferred from microsatellite data using STRUCTURE version 2.3.3 (Pritchard et al., 2000). STRUCTURE analysis implements a Bayesian clustering algorithm to assign genotypes to clusters that minimize Hardy–Weinberg disequilibrium and linkage disequilibrium. Fifteen replicate runs were conducted for each K between 1 and 10 without prior information using the admixture model and assuming correlated allele frequencies (Falush et al., 2003). Each run consisted of 1,000,000 Markov chain Monte Carlo (MCMC) replications after burn-in with 100,000 iterations. Optimal K was determined using the ΔK method of Evanno et al. (2005), as implemented in STRUCTURE HARVESTER (Earl and vonHoldt, 2012). Outputs of STRUCTURE analyses were visualized using the software CLUMPP (Jakobsson and Rosenberg, 2007) and DISTRUCT (Rosenberg, 2004).

#### Detection of Contemporary Gene Flow

To detect the direction and rates of contemporary migration among populations of *S. alterniflora* in China and America, the program BAYESASS version 3.0.4 (Wilson and Rannala, 2003) was used. BAYESASS uses a Bayesian approach and an MCMC algorithm to estimate migration rates (m) between populations without assuming genetic equilibrium, which reflects gene flow over the several most recent generations (Wilson and Rannala, 2003). Preliminary runs were performed to adjust the MCMC mixing parameters of migration rates (*m*), allele frequencies (*a*), and inbreeding coefficients (*f*), in order to reach a swap acceptance rate between 20 and 60%, as suggested by Wilson and Rannala (2003). For populations from China and the United States, the mixing parameters *m* = 0.15, *a* = 0.4, and *f* = 0.5 were used. Six independent replicates with different random starting seeds were performed to check for convergence for each analysis with a burn-in 50,000,000 iterations followed by 5,000,000 MCMC iterations and a sampling interval of 2,000. Convergence of the MCMCs was checked by comparing the traces of each run using TRACER version 1.7 (Rambaut et al., 2018). Among the six independent runs, the one with the best consistency of the posterior distribution was chosen.

## Results

### Genetic Diversity

The mean number of alleles per locus (*A*) and allelic richness (*A*_R_) in each sampling site ranged from 2.125 (LN) to 9.125 (GA_2_), and 2.014 (LN) to 7.855 (GA_2_), respectively. Observed and expected heterozygosity (*H*_O_ and *H*_E_) in each sampling site ranged from 0.270 (LN) to 0.885 (GD), and 0.224 (LN) to 0.725 (GA_2_), respectively. Inbreeding coefficients (*F*_IS_) ranged from -0.957 (GD) to 0.296 (GA_2_) (mean value 0.063), and inbreeding coefficients were significant. From the data, the lowest and highest diversity populations were LN and GA_2_ respectively (Table 2).

The mean numbers of alleles per locus (*A*) were 9.750 and 13.125; allelic richness (*A*_R_) values were 9.666 and 13.007; observed heterozygosity (*H*_O_) values were 0.538 and 0.530, and expected heterozygosity (*H*_E_) values were 0.725 and 0.744 for the Chinese invasive and the American native groups, respectively (Table 3). The mean number of alleles per locus (*A*) and allelic richness (*A*_R_) of the Chinese invasive group (*A* = 9.750, *A*_R_ = 9.666) were both lower than those of the American native group (*A* = 13.125, *A*_R_ = 13.007). However, observed and expected heterozygosity (*H*_O_ and *H*_E_) of the Chinese invasive group (*H*_O_ = 0.538, *H*_E_ = 0.725) were similar with those of the American native group (*H*_O_ = 0.530, *H*_E_ = 0.744) (Table 3).

### Genetic Differentiation Among Populations

ARLEQUIN version 3.5 was used to detect considerable genetic differentiation, both among and within populations (among and within individuals) within a given region. In the Chinese invasive group, the proportion of genetic variance was significantly partitioned among populations (30.19%; Table 4). In the American native group, the proportion of genetic variance was significantly partitioned among populations (10.28%; Table 4). Genetic differentiation among the Chinese invasive populations (*Fst* = 0.30193; Table 4) was significantly stronger than among the American native populations (*Fst* = 0.10284; Table 4).

**TABLE 4:** Analysis of molecular variance showing degrees of freedom (d.f.), sum of squares (SS), variance components (Var.), percentage of variances (%).

Source of variation	d.f.	SS	Var.	%	F statistics	F
**(A) Chinese populations of spartina alterniflora**						
Among populations	8	550.162	0.88006	30.19	*F*_*IS*_	–0.04295
Within populations	335	652.343	–0.08740	–3.00	*F*_*ST*_FST	0.30193
Within individuals	334	730.000	2.12209	72.81	*F*_*IT*_FIT	0.27195
Total	687	1, 932.506	2.91475			
**(B) American populations of *s. alterniflora***						
Among populations	8	129.760	0.17576	10.28	*F*_*IS*_FIS	0.12890
Within populations	362	626.564	0.19763	11.56	*F*_*ST*_FST	0.10284
Within individuals	371	495.500	1.33558	78.15	*F*_*IT*_FIT	0.21849
Total	741	1251.825	1.70897			

In the Chinese invasive group, pairwise *F*_ST_ values ranged from 0.04418 (ZJ and GX) to 0.56815 (LN and GD). *F*_ST_ was statistically significant between all population pairs (Table 5). Among the three populations in the American native group, pairwise *F*_ST_ values ranged from 0.03518 (NC and GA) to 0.06871 (GA and FL), and *F*_ST_ was statistically significant between all population pairs (Table 5).

**TABLE 5:** Genetic differentiation between populations of Spartina alterniflora at eight microsatellite loci.

(A)										

	LN	HB	SD	JS		ZJ	FJ	XG	GD	GX
LN		*	*	*	*	*	*	*	*	*
HB	0.35113		*	*	*	*	*	*	*	*
SD	0.56222	0.26527		*	*	*	*	*	*	*
JS	0.47240	0.15118	0.30227		*	*	*	*	*	*
ZJ	0.31917	0.12467	0.32710	0.25278		*	*	*	*	*
FJ	0.41437	0.08146	0.27577	0.16666	0.13817		*	*	*	*
XG	0.48148	0.28666	0.47472	0.39842	0.28518	0.34836		*	*	*
GD	0.56815	0.27143	0.49464	0.33050	0.42084	0.33986	0.44268		*	*
GX	0.24262	0.06302	0.24355	0.17463	0.04418	0.05575	0.22705	0.31109		*

**(B)**										

	**NC**	**GA**	**FL**							

NC		*	*							
GA	0.03518		*							
FL	0.06166	0.06871								

Fst below the diagonal with the level of significance above the diagonal. *P-values significant at (P < 0.05). Analyses performed with Arlequin software.

However, the Mantel test results indicated that there were no significant correlations (*R*^2^ = 5E-05, *P* = 0.489, invasive populations; *R*^2^ = 0.0341, *P* = 0.114, native populations) between the genetic differentiation index (*F*_ST_) and geographic distance (in kilometers) among both the invasive and native populations (Supplementary Figure S1).

### Population Genetic Structure

**FIGURE 2:**
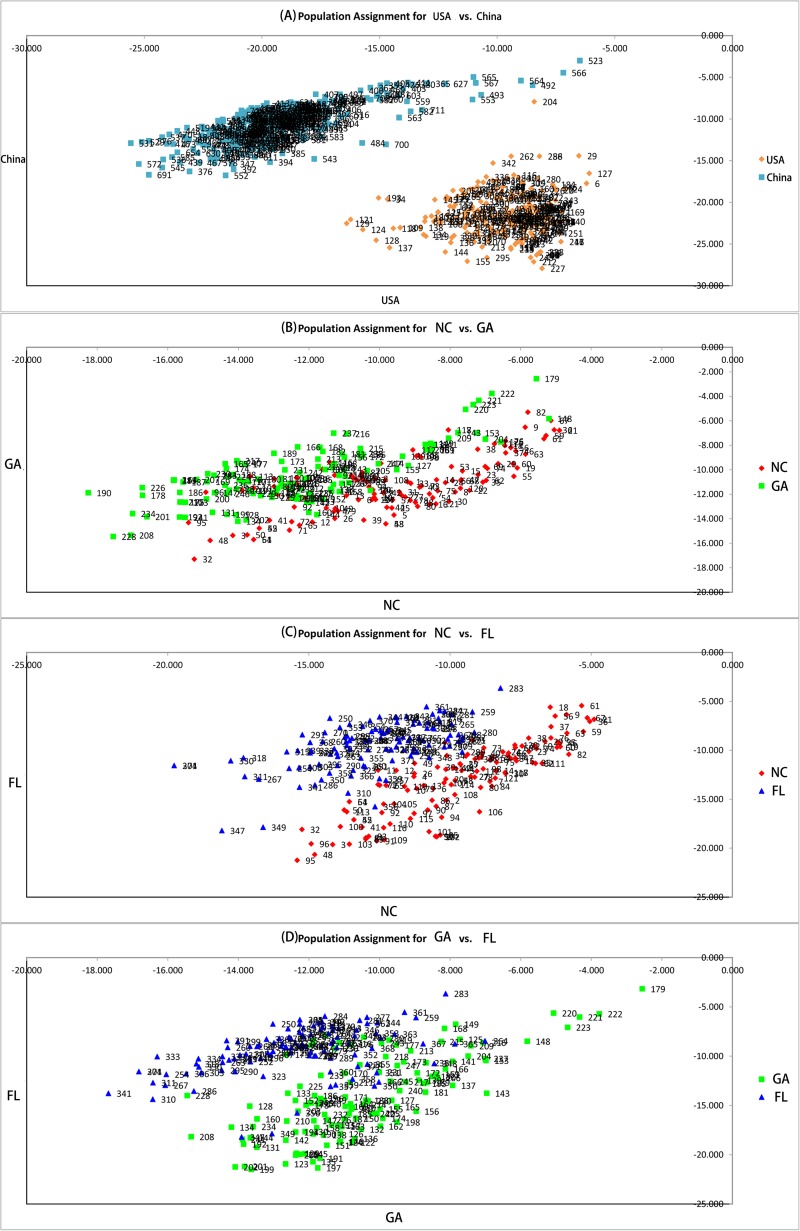
Pairwise population assignment. **(A)** Pairwise population assignment between American and Chinese populations of *Spartina alterniflora*. **(B)** Pairwise population assignment between populations of *S. alterniflora* in North Carolina and Georgia. **(C)** Pairwise population assignment between populations of *S. alterniflora* in North Carolina and Florida. **(D)** Pairwise population assignment between populations of *S. alterniflora* in Georgia and Florida.

**FIGURE 3:**
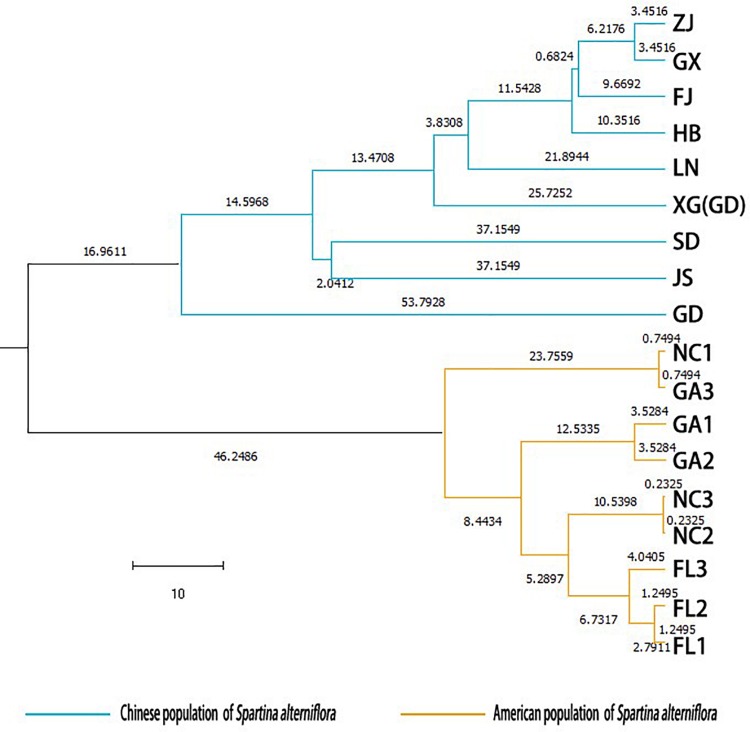
Unweighted pair-group method with arithmetic average dendrogram based on Nei’s (1973) genetic distance among the populations of *Spartina alterniflora.*

**FIGURE 4:**
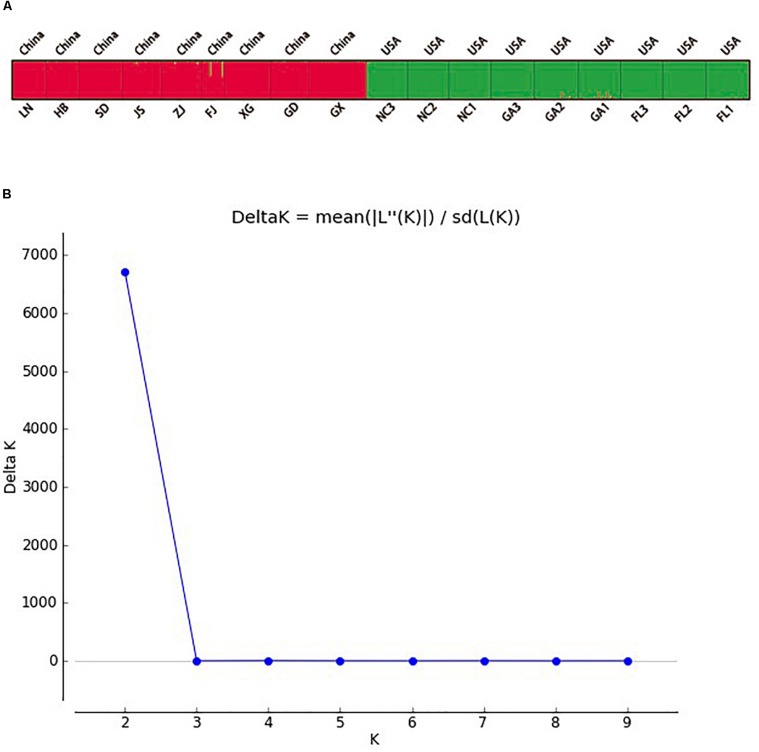
The population genetic structure of *Spartina alterniflora* across invasive and native ranges **(A)** and the Delta K analysis **(B)**.

Two principal clusters were indicated, and high degrees of genetic differentiation between the Chinese and American groups were apparent in population assignments. All Chinese populations were separated from all American sampling sites (Figure 2). Unweighted pair-group method with arithmetic average also showed that all the Chinese populations and all of the American sampling sites clustered separately in two different clusters (Figure 3). The results of the STRUCTURE analysis, based on Bayesian statistical model-based clustering, indicated the existence of obvious population genetic structure among all sampling sites (Figure 4). When *K* = 2, the value of ΔK was largest [mean Ln P(D) = -18,951.46, Δ*K* = 6,712.51; Figure 4]. When *K* = 2, the nine populations sampled from China were clustered as being genetically similar, and the nine populations sampled from America were clustered as being genetically similar, which was the same result shown by the population assignment and UPGMA (Figures 2, 3).

**FIGURE 5:**
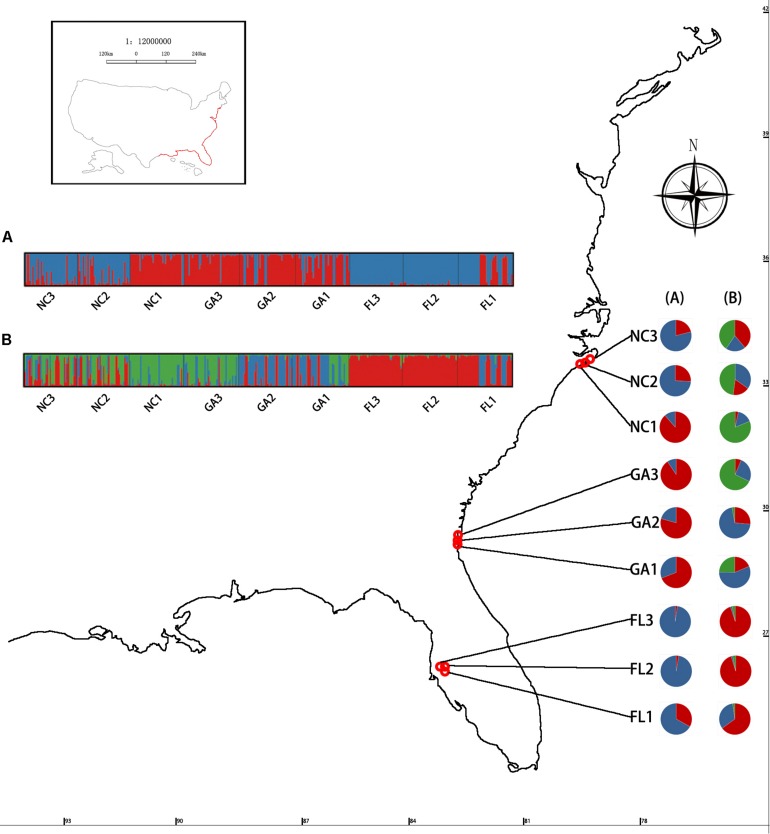
Geographic distribution of the genetic groups on the coast of America detected using STRUCTURE analysis of *Spartina alterniflora* based on SSR data. *K*-values of the panel Δ*K* = 2 **(A)** and Δ*K* = 3 **(B)**.

High degrees of genetic differentiation among the three populations (NC, GA, and FL) in the American native group were apparent in population assignment. The result of the UPGMA showed that NC_1_ and GA_3_ clustered together, two populations (NC_2_ and NC_3_) sampled from North Carolina clustered together, two populations (GA_1_ and GA_2_) sampled from Georgia clustered together, and three populations (FL_1_, FL_2_, and FL_3_) from Florida clustered together (Figure 3). The results of the STRUCTURE analysis, based on Bayesian statistical model-based clustering, indicated the existence of obvious population genetic structure among nine populations sampled from America. When *K* = 2, the value of ΔK was largest [mean Ln P(D) = -9,404.57, Δ*K* = 870.65; Figure 5]. When *K* = 3, although there was genetic admixture among the nine American populations, the results were basically consistent with those obtained using UPGMA.

**FIGURE 6:**
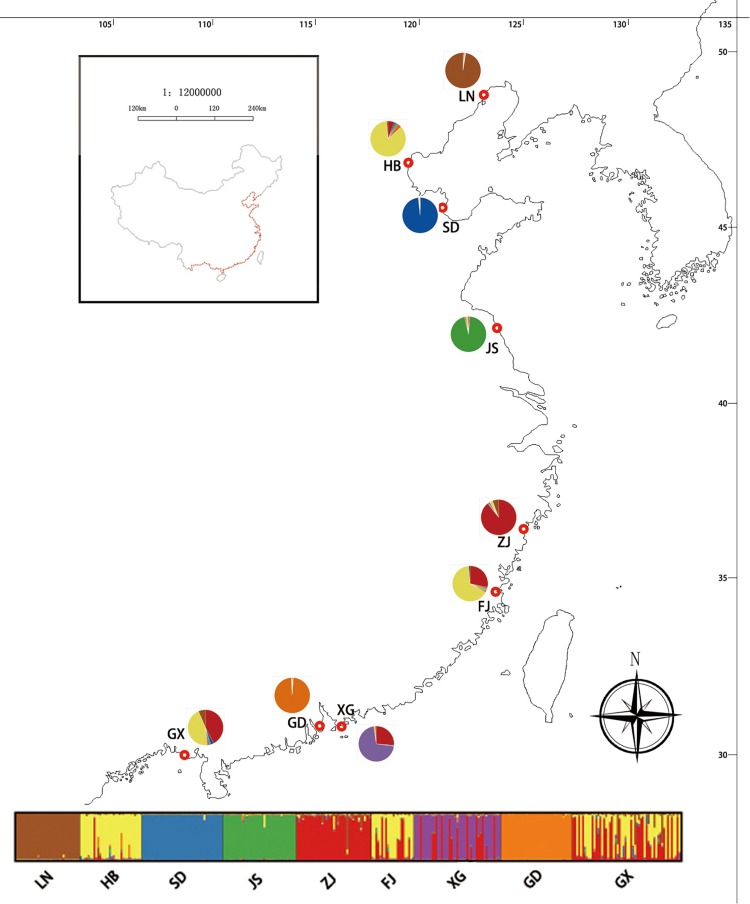
Geographic distribution of the genetic groups on the coast of China detected using STRUCTURE analysis of *Spartina alterniflora* based on SSR data (Δ*K* = 7).

The results of the STRUCTURE analysis, based on Bayesian statistical model-based clustering, indicated the existence of obvious population genetic structure among the nine populations sampled from China. When *K* = 2, the value of ΔK was largest [mean Ln P(D) = -18,951.46, Δ*K* = 6,712.51; Figure 6]. Most Chinese populations form separate clusters, with only three populations (HB, FJ, and GX) having admixture, and similar genetic structure (Figure 6).

### Gene Flow

The results estimated by the BAYESASS analysis showed the average recent migration rates in pairwise comparisons of the nine Chinese populations (m_c_) ranged from 0.0051 (JS to GX; GD to GX; *P* < 0.05) to 0.2265 (GX to FJ; *P* < 0.05), and the average recent migration rates in pairwise comparisons of three American populations from North Carolina, Georgia, and Florida (m_a_), ranged from 0.0041 (NC to FL; *P* < 0.05) to 0.1230 (NC to GA; *P* < 0.05). The lowest and highest values of contemporary gene flow in pairwise comparisons among the populations from China were both higher than those among the populations from the United States.

**FIGURE 7:**
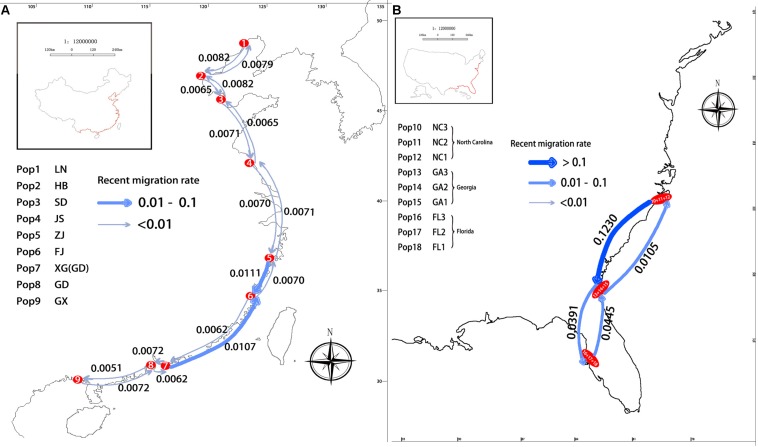
Recent migration rate between pairs of neighboring populations **(A)** in China; **(B)** in United States.

However, the maximum gene flow of the nine Chinese populations was found between GX and FJ, which are not neighboring populations. The results estimated by the BAYESASS analysis also showed that the average recent migration rates in comparison between neighboring populations of China ranged from 0.0051 (GD to GX, *P* < 0.05) to 0.0111 (ZJ to FJ, *P* < 0.05) (Figure 7A). The lowest and highest values of contemporary gene flow in comparisons between neighboring populations of China were both lower than those between neighboring populations from the United States, which ranged from 0.0105 (GA to NC; *P* < 0.05) to 0.1230 (NC to GA; *P* < 0.05) (Figure 7B).

Contemporary gene flow revealed asymmetric gene flow in *S. alterniflora* along the coastline of China and the United States, which were congruent with the direction of seasonal marine currents (Figure 7). For populations along the South China Sea coastline (e.g., population XG and FJ), strong gene flow was detected from south to north, almost two times larger than that in the opposite direction (Figure 7A), which was congruent with the direction of the South China Sea Warm Current. Analogously, for populations along the East China Sea coastline, strong asymmetric gene flow was detected from population ZJ to FJ (from north to south), which is congruent with the direction of the China Coastal Current (Figure 7A). A similar phenomenon has been observed in populations of *S. alterniflora* from the United States. Highly asymmetric gene flow was detected from north to south (from population NC to GA), almost 11 times larger than that in the opposite direction, which is congruent with the result of the STRUCTURE analysis that there is genetic mixture between the NC and GA populations (Figure 7B).

## Discussion

### The Invasive Chinese *S. alterniflora* Populations Have Completely Differentiated From Their Native Congeners

Our study shows that the *S. alterniflora* populations on the coast of China have completely differentiated from their native congeners. Qiao et al. (2019) also considered that there was a trend of differentiation between the invasive populations of China and the native populations of America. Genetic divergence between the invasive and original populations may be because of either hybridization after multi-invasion or the postintroduction rapid evolution, or both.

In England (Southampton Bay), *S. alterniflora* hybridization with *Spartina maritima* resulted in a sterile hybrid *Spartina* × *townsendii*, and chromosome doubling in this hybrid gave rise to a new fertile allopolyploid species, *Spartina anglica* (Ainouche et al., 2003). *Spartina alterniflora* in Willapa Bay, USA, was considered to be the offspring of hybridization among previously non-overlapping populations, which caused the *S. alterniflora* population in Willapa Bay to diverge from its native Atlantic coast counterparts (Blum et al., 2007). *Spartina alterniflora* in San Francisco Bay, USA, was a very rare *S. alterniflora* of haplotype D, which was considered to be the offspring of hybridization of *S. alterniflora* from the Atlantic coast with a native congener (Blum et al., 2004, 2007). Other invasive species have also shown similar cases. For example, *Phragmites australis*, a cryptic invasive from North America, which was very aggressive, is a hybrid between the European *P. australis* of haplotype M and a North American native congener (Sattonstall, 2002; Paul et al., 2010). All seedlings and seeds collected from the United States were first planted in Luoyuanwan, Fujian Province, in 1981, and then were cultivated separately according to different sources, and introductions in different coastal areas of China were different. However, this species is an anemophilous plant (Wang et al., 2006), so it was not possible to determine whether cross-breeding occurred between plants of different origins during this period. Qiao et al. (2019) suggested that hybridization during this period contributed to the differentiation of Chinese *S. alterniflora* from its American origins.

The postintroduction rapid evolution also may enable invasive species to better adapt to novel environments in the introduced range (Broennimann et al., 2007; Beaumont et al., 2009; Gallagher et al., 2010; Xu et al., 2015). For example, Beaton et al. (2011) found that invasive genotypes of *Lespedeza cuneata* are more competitive than either the ancestral or native genotypes. Liu et al. (2019) also found Chinese *S. alterniflora* plants were taller and denser and set up to four times more seed than US plants in both the field and common garden, and they considered that invasive plants evolved rapidly in the new range.

### Genetic Structure of the Invasive Populations in China

The loss of genetic variation may reduce the evolutionary potential of the population (Allendorf, 1986). There are several ways to measure genetic variation; however, the two most considerations of this topic use heterozygosity and allelic diversity (Nei et al., 1975; Sirkkomaa, 1983; Allendorf, 1986). In the study, the mean number of alleles per locus (*A*) and allelic richness (*A*_R_) of *S. alterniflora* from the coast of China was lower than those of the native *S. alterniflora*, which is the same finding as previous research by Deng et al. (2007), Gong et al. (2014), and Xia et al. (2015) (Table 3). However, observed and expected heterozygosity (*H*_O_ and *H*_E_) of the *S. alterniflora* from the Chinese invasive group was similar to those of the native *S. alterniflora* from the American native group (Table 3). This may be due to the founder effect, the bottleneck (Nei et al., 1975; Allendorf, 1986; Dlugosch and Parker, 2008b; Hundertmark and Van Daele, 2010), and admixture. Bottlenecks often have a greater effect on the number of alleles present than on heterozygosity when there are multiple alleles present (Allendorf, 1986). Therefore, heterozygosity may provide an overly optimistic view when there are many alleles at a locus or when the population goes through a small bottleneck (Allendorf, 1986). Meanwhile, low-frequency alleles in populations are determined by the initial allelic diversity (Allendorf, 1986). The founding of a new population in a new habitat often entails a reduction in population size (Santos et al., 2012), and the initial allelic diversity of *S. alterniflora* of human-mediated introductions in China must be finite.

**FIGURE 8:**
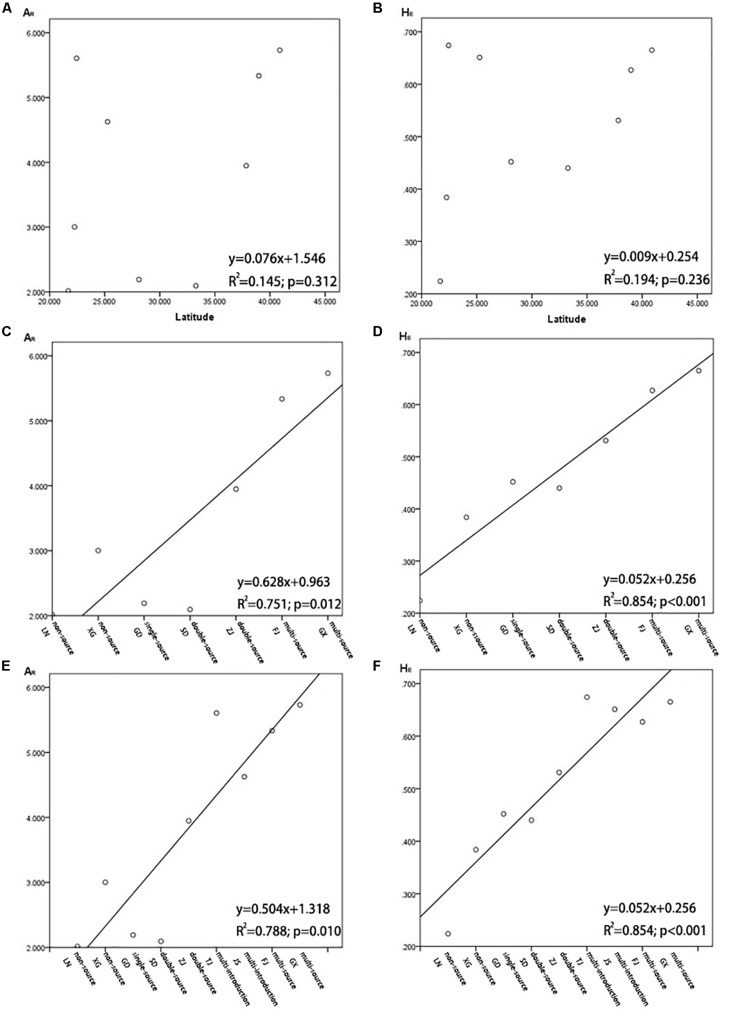
**(A)** Population allelic richness (*A*_R_) in relation to latitude of *Spartina alterniflora* in China; **(B)** population expected heterozygosity (*H*_E_) in relation to latitude of *S. alterniflora* in China; **(C)** population allelic richness (*A*_R_) in relation to the number of sources when *S. alterniflora* was introduced into coastal China for the first time (1980s), and it is not including HB and JS; **(D)** population expected heterozygosity (*H*_E_) in relation to the number of sources when *S. alterniflora* was introduced into coastal China for the first time (1980s), not including HB and JS; **(E)** population allelic richness (*A*_R_) in relation to the number of sources when *S. alterniflora* was introduced into coastal China for the first time (1980s), including HB and JS; **(F)** population expected heterozygosity (*H*_E_) in relation to the number of sources when *S. alterniflora* was introduced into coastal China for the first time (1980s), including HB and JS.

A series of genetic studies have found evidence of latitudinal changes in *S. alterniflora* (Seneca, 1974; Freshwater, 1988). Somers and Grant (1981) considered that there was a strong correlation between geographical range and genetic differentiation of *S. alterniflora* on the Atlantic coast of the United States, and O’Brien and Freshwater (1999) and Travis and Hester (2005) reported the same. However, our results showed that there was no correlation of genetic parameters (*A*_R_ and *H*_E_) and geographic coordinates (latitudes) detected in the nine invasive populations on the coast of China (*R*^2^ = 0.145, *P* = 0.312; *R*^2^ = 0.194, *P* = 0.236; Figure 8). There also was not a significant correlation between the genetic differentiation index (*F*_ST_) and geographic distance (in km) among the invasive populations (Supplementary Figure S1). This result is consistent with our previous research (Xia et al., 2015).

The distribution of invasive species is continuous at a small scale, but it might not be continuous at a large scale. *Diabrotica virgifera*, which is native to Mexico, has been found in several isolated populations in its introduced range in China. However, the genetic information of the original populations and the invasive populations of *D. virgifera* were analyzed by molecular markers, and these isolated invasive populations were directly related to their original populations across the Atlantic (Miller et al., 2005; Ciosi et al., 2008). Blum et al. (2007) suggested that the varying levels of genetic diversity among the invasive populations in the United States correspond to known or hypothesized differences in invasion history. Therefore, we analyzed the correlation between the genetic parameters (*A*_R_ and *H*_E_) and the number of sources of each population on the coast of China in the historical reports when they were introduced in the 1980s. Three natural populations from the United States were thought to be divergent, and our study showed that there was significant differentiation among populations from North Carolina, Georgia, and Florida, except for the overlap of genotypes between the sampling sites of NC_1_ and sites of GA_3_ (Figures 2, 3). This means that the more sources of *S. alterniflora* that were introduced into a population of China in the 1980s, the more genotypes of *S. alterniflora* were likely to be introduced. Our analysis proved this hypothesis. However, because the HB population and the JS population were introduced many times in 1980s, and we did not find sources of the HB and JS populations in each introduction, we first excluded the HB and JS populations. Other populations with the same number of introduced sources were placed into the same clusters: non-introduction, single-source introduction, double-source introduction, multisource introduction. Populations in the four groups arranged from north to south. The results showed that there was a positive correlation of the genetic parameters (*A*_R_ and *H*_E_) of *S. alterniflora* and the number of sources (Figure 8). Because of there being multiple introductions both in the HB population and JS population, we combined the multi-introduction with the multisource populations, and the populations in these grouping were still listed from north to south. The results showed that the genetic parameters (*A*_R_ and *H*_E_) of *S. alterniflora* and the number of sources were also positively correlated (Figure 8). Expected heterozygosity (*H*_E_ = 0.2804, 0.2799, 0.2969, 0.3223, 0.3298, 0.2525, 0.3238) in our previous research (Xia et al., 2015), which we checked in samples from seven populations from China [TJ (HB), SD, JS, ZJ, FJ, GD, GX] and measured using the AFLP method, was analyzed using the same approach mentioned above. The genetic parameters (*H*_E_) of *S. alterniflora* and the number of sources were also positively correlated (*R*^2^ = 0.597, *P* < 0.05; Xia et al., 2015).

Our results are consistent with introduction records of *S. alterniflora* on the coast of China in the 1980s. The populations that were composed of individuals from different sources had higher diversities than the populations that were composed of individuals from a single source (Dlugosch and Parker, 2008a). Another invasive population of *S. alterniflora* in San Francisco Bay also showed high genetic diversity due to hybridization after multi-invasion (Blum et al., 2007).

In addition, we also found that the LN, HB, SD, ZJ, FJ, and XG population in China all showed the minimum differentiation from the GX populations (Table 5), and the JS and GD populations both had the minimum differentiation from the HB populations (Table 5). The LN, ZJ, and GX population all had the maximum differentiation from the GD population (Table 5). The HB, SD, JS, FJ, XG, and GD population all had the maximum differentiation from the LN population (Table 5). In the Chinese invasive group, all populations had the minimum differentiation from the GX, FJ, or HB population, which were multiple sources or multiple introductions during the human-mediated introductions in the 1980s. All populations had the maximum differentiation from the LN or GD population, which were non-introduction or single-introduction populations. This might be because the GX, FJ, and HB populations contained the maximum number of genotypes introduced from the original populations; that is, these three populations might have contained all the genotypes of the other populations that were introduced in the 1980s. In contrast, the LN and GD population contained fewer genotypes that were introduced from the original populations; that is, individual genotypes introduced into other populations might be neither or rarely introduced into the LN and GD population. Moreover, we observed that the maximum gene flow pairwise comparison among the nine populations from China was higher than that among the three populations from America. However, the maximum gene flow among the nine populations from China was not between two neighboring populations (Supplementary Table S2). The results of UPGMA and STRUCTURE also showed less differentiation among HB, ZJ, FJ, and GX than that among other populations, and admixture was found among these four populations (Figures 3, 5). Therefore, we consider that the genetic structure of *S. alterniflora* along the coast of China was mainly influenced by human-mediated introductions.

The HB population had a high genetic diversity and a similar genetic composition with the FJ and GX populations, so we considered that all three sources of *S. alterniflora* had been introduced into the HB population.

Moreover, Liu et al. (2016, 2017) found low fecundity of low-latitude *S. alterniflora* populations in China, and a slower rate of spread at low latitudes than at high latitudes in China. Wang et al. (2012) found there were significant positive relationships between genotypic diversity and maximum spread distance, patch size, shoot number per patch, and aboveground biomass. In addition, Qiao et al. (2019) observed that individuals of *S. alterniflora* in the south population (including one Fujian populations and two populations in Guangdong) showed a weaker competitiveness than individuals of *S. alterniflora* in the northern population (the Tianjin and Shandong population) and the middle population (the Jiangsu, Shandong, Zhejiang, and Fujian population). It is universally recognized that the evolutionary potential of a population is proportional to its amount of genetic diversity (Vilas et al., 2015). However, Qiao et al. (2019) did not observe significant neutral genetic differentiation among several populations, but they considered that there was significant evolution of *S. alterniflora* in the northern and middle population, whereas individuals of the southern population did not undergo significant evolution and selection. According to this study, it might be because individuals of *S. alterniflora* that were introduced into GD in the 1980s were from a single source (Florida), causing a lower genetic diversity, thereby limiting the evolutionary potential (Xu et al., 2015). In fact, hybridization after multi-invasion is a powerful force in the evolution of plants (Birchler et al., 2003). Introduction from a single source may also cause a lack of heterosis and weak competitiveness of *S. alterniflora* in the GD population. However, to test our hypothesis, another control experiment needs to be added. The GX population was also located in southern China, and individuals of *S. alterniflora* in the GX population should be compared with individuals of other populations, because *S. alterniflora* individuals that were introduced into GX in the 1980s were from multiple sources that had similar genetic components with those in the FJ and HB population (Figure 5).

### Genetic Differentiation Among the Invasive Populations From China

Pairwise *F*_ST_ values of the Chinese invasive group covered a large range, which indicated that among some populations of *S. alterniflora* in China, there might be substantial differentiation (Tables 4, 5). Zhang et al. (2008) found that 12 of the 17 traits of *S. alterniflora* in 10 populations from China (from TJ to GD) were significantly different, and the mean flowering date and the relative growth rate showed significant latitudinal variation. *Spartina alterniflora* from different populations maintained their morphological and physiological traits in the common garden experiment. Although Qiao et al. (2019) did not observe differentiation in neutral genetics, strong quantitative genetic divergence was found among invasive populations. However, we observed a strong neutral genetic divergence among nine invasive populations in China, which was greater than that observed among the three native populations in the United States (Tables 4, 5). This might be because of the different microsatellite loci we selected, or because they collected only 10 samples in each population, which might not adequately reflect the invasive populations.

O’Brien and Freshwater (1999) and Blum et al. (2007) considered that there was a low gene flow among populations of *S. alterniflora* on the Atlantic coast and the Gulf of North America through using RAPD marker analysis, cpDNA analysis, and microsatellites analysis. *Spartina alterniflora* is distributed from Freeport, Marine (43°N) to Hollywood, Florida (27°N) on the Atlantic coast (Kirwan et al., 2009). O’Brien and Freshwater (1999) and Blum et al. (2007) considered that the low gene flow among populations of *S. alterniflora* on the Atlantic coast of the United States was due to subtle selection pressure provided by environmental stresses along the latitudinal gradient.

We also observed that the minimum and maximum gene flow between pairs of neighboring populations in China were lower than those between pairs of neighboring populations in the United States (Figure 7). *Spartina alterniflora* is distributed from Liaoning (41°N) to Guangxi (20°N) on the coast of China (Kirwan et al., 2009), and there are significant differences in environments and flowering phenologies among populations from Liaoning to Guangxi on the coast of China (Zhang et al., 2008). Therefore, we considered that, excluding personal factors, the naturally occurring gene flow of *S. alterniflora* on the coast of China could not be high, especially between populations that are far apart, and the differentiation among populations due to limited gene exchange and different selection forces. In fact, the human-mediated introduction strongly affected the gene flow among the Chinese invasive populations, because we observed the maximum gene flow was between FJ and GX, and Guangdong Province is in the area between Fujian and Guangxi Province; however, the gene flow between GD and FJ, and GD and GX, was much lower than between FJ and GX (Figure 7). So, we also considered that the low natural gene flow among the invasive populations in China caused genetic structure of the invasive populations that was still affected by human-mediated introductions (Slatkin, 1987). The low gene flow among populations of *S. alterniflora* on a large scale would cause the isolation of different populations from each other, which might further promote the differentiation of these populations, such as the significant differentiation that occurred between the populations on the Atlantic coast and on the Gulf of the United States (Seneca, 1974; O’Brien and Freshwater, 1999; Blum et al., 2007).

## Conclusion

Because of admixture and intraspecies hybridization involving secondary contact after invasion, or rapid evolution of invasive plants after they colonize new ranges, invasive *S. alterniflora* populations from China have completely differentiated from that of the native populations from the United States. Similar heterozygosity of the Chinese invasive and American native populations, but lower allelic diversities of the Chinese invasive populations, may be due to the founder effect, or the bottleneck, which supports the theory that the allelic diversity is more sensitive to population bottlenecks than heterozygosity. We also found a positive correlation of the genetic diversity of *S. alterniflora* and the number of sources of each population along the coast of China, which is a strong evidence to show that multiple introduction can even increase genetic diversity in the introduced range. The higher genetic diversities and the rapider spread of this species at high latitudes than at low latitudes in China is a strong evidence to show that multiple introduction facilitates invasion ability of *S. alterniflora*. Because of the low natural gene flow among the invasive populations in China, the genetic structure of invasive populations in China was still influenced by the human-mediated introductions 40 years ago. There was significant differentiation among invasive populations, which were mainly affected by human mediated introductions in the 1980s and may be due to isolation among the invasive populations and different selection forces.

The human-mediated long-distance dispersal should take the most of responsibility for the rapid spread of *S. alterniflora* along the coast of China. Multisource introductions of *S. alterniflora* are perhaps helpful for local adaptation but itself cannot cause rapid spread along the whole coast of China.

Meanwhile, in the future, the prevention of gene exchange, whether manmade or natural, among populations of *S. alterniflora* is the first and most important step in the control of the species on the coast of China, because admixture and hybridization of isolated populations may generate new heterosis and increase the difficulty of managing *S. alterniflora* in China.

Neutral molecular data can help us to understand the rapid evolution, isolation, and the divergence of a population, however, they can provide only partial insight into adaptation of a population to a new environment. Therefore, in the future studies, molecular data and trait data should be combined, yielding more powerful evolutionary studies.

In addition, no matter how much we lose from invasive organisms, at least, they lead to unanticipated scenarios to understand the natural world better across large spatial and temporal scales, such as extinction, ecosystem function, and the response of species to climate change. They also provide opportunities to observe ecological and evolutionary processes in real-time and to quantify rate processes, such as genetic change, which is very difficult to observe with native species. We highlight the utility of using *S. alterniflora* as the “model organism” in studying the evolution of plants, especially the invasive populations of *S. alterniflora* on the coast of China. We intend to assess how an alien species evolves from the inhomogeneous distribution (genetic structure) to a uniform distribution (genetic distance was positively correlated with geographic distance) in its invasive region (the coast of China) across a large geographic scale through regular observation of *S. alterniflora*.

## Data Availbaility Statement

The datasets generated by this study are available on request to the corresponding author.

## Author Contributions

LX set up most of the experimental plots, analyzed most of the data, ran the statistical models, created the graphics, and wrote the first draft of the manuscript. QG developed the research questions, designed the experiments, set up a part of the experimental plots, and analyzed a part of the data. All authors contributed to the manuscript revision, read and approved the submitted version.

## Conflict of Interest

The authors declare that the research was conducted in the absence of any commercial or financial relationships that could be construed as a potential conflict of interest.

## References

[B1] Ainouche M. L. Baumel A. Salmon A. Yannic G. (2003). Hybridization, polyploidy and speciation in *Spartina* (Poaceae). *New Phytol.* 161 165–172. 10.1046/j.1469-8137.2003.00926.x

[B2] Allendorf F. W. (1986). Genetic dift and the loss of alleles versus heterozygosity. *Zoo Biol.* 5 181–190. 10.1002/zoo.1430050212

[B3] An S. Q. Gu B. H. Zhou C. F. Wang Z. S. Deng Z. F. Zhi Y. B. (2007). *Spartina* invasion in China: implications for invasive species management and future research. *Weed Res.* 47 183–191. 10.1111/j.1365-3180.2007.00559.x

[B4] Beaton L. L. Van Zandt P. A. Esselman E. J. Knight T. M. (2011). Comparison of the herbivore defense and competitive ability of ancestral and modern genotypes of an invasive plant *Lespedeza cuneate*. *Oikos* 120 1413–1419. 10.1111/j.1600-0706.2011.18893.x

[B5] Beaumont L. J. Gallagher R. V. Thuiller W. Downey P. O. Leishman M. R. Hughes L. (2009). Different climatic envelopes among invasive populations may lead to underestimations of current and future biological invasions. *Divers. Distrib.* 15 409–420. 10.1111/j.1472-4642.2008.00547.x

[B6] Birchler J. A. Auger D. L. Riddle N. C. (2003). In search of the molecular basis of heterosis. *Plant Cell* 15 2236–2239. 10.1105/tpc.151030 14523245 PMC540269

[B7] Blakeslee A. M. H. Manousaki T. Vasileiadou K. Tepolt C. K. (2019). An evolutionary perspective on marine invasions. *Evol. Appl.* 13:12906 10.1111/eva.12906 PMC7045714 32431730

[B8] Blum M. J. Bando K. J. Katz M. Strong D. R. (2007). Geographic structure, genetic diversity and source tracking of *Spartina alterniflora*. *J. Biogeogr.* 34 2055–2069. 10.1111/j.1365-2699.2007.01764.x

[B9] Blum M. J. Sloop C. M. Ayres D. R. Strong D. R. (2004). Characterization of microsatellite loci in *Spartina species* (Poaceae). *Mol. Ecol. Notes* 4 39–42. 10.1046/j.1471-8286.2003.00556.x 25202479

[B10] Broennimann Q. Treier U. A. Muller-Scharer H. Thuiller W. Peterson A. T. Guisan A. (2007). Evidence of climatic niche shift during biological invasion. *Ecol. Lett.* 10 701–709. 10.1111/j.1461-0248.2007.01060.x 17594425

[B11] Chen J. Q. Chung C. H. (1990). Differentiation of three ecotypes in responses to salt stress in *Spartina alterniflora*. *Acta Phytoecol. Geobot. Sin.* 14 33–39.

[B12] Ciosi M. Miller N. J. Kim K. S. Giordano R. Estoup A. Guillemaud T. (2008). Invasion of Europe by the western corn rootworm, *Diabrotica virgifera*: multiple transatlantic introductions with various reductions of genetic diversity. *Mol. Ecol.* 17 3614–3627. 10.1111/j.1365-294X.2008.03866.x 18662220

[B13] Civille J. C. Sayce K. Smith S. D. Strong D. R. (2005). Reconstructing a century of *Spartina* invasion with historical records and contemporary remote sensing. *Ecoscience* 12 330–338. 10.2980/i1195-6860-12-3-330.1

[B14] Deng Z. F. An S. Q. Zhi Y. B. Zhou C. F. Chen L. Zhao C. J. (2006). Preliminary studies on invasive model and outbreak mechanism of exotic species, *Spartina alterniflora* loisel. *Acta Ecol. Sinic.* 26 2678–2686.

[B15] Deng Z. F. An S. Q. Zhou C. F. Wang Z. S. Zhi Y. B. Wang Y. J. (2007). Genetic structure and habitat selection of the tall form *Spartina alterniflora* Loisel. in China. *Hydrobiologia* 2007 195–204. 10.1007/s10750-006-0529-x

[B16] Dlugosch K. M. Parker I. M. (2008a). Founding events in species invasions: genetic variation, adaptive evolution, and the role of multiple introductions. *Mol. Ecol.* 17 431–449. 10.1111/j.1365-294X.2007.03538.x 17908213

[B17] Dlugosch K. M. Parker I. M. (2008b). Invading populations of an ornamental shrub show rapid life history evolution despite genetic bottlenecks. *Ecol. Lett.* 11 701–709. 10.1111/j.1461-0248.2008.01181.x 18410377

[B18] Doyle J. J. Doyle J. L. (1987). A rapid DNA isolation procedure for small quantities of fresh leaf tissue. *Phytoche. Bull.* 19 11–15.

[B19] Earl D. A. vonHoldt B. M. (2012). Structure harvester: a website and program for visualizing structure output and implementing the evanno method. *Conserv. Genet. Resour.* 4 359–361. 10.1007/s12686-011-9548-7

[B20] Ellegren H. (2004). Microsatellites: simple sequences with complex evolution. *Nat. Rev. Genet.* 5 435–445. 10.1038/nrg1348 15153996

[B21] Evanno G. Regnaut S. Goudet J. (2005). Detecting the number of clusters of individuals using the software structure: a simulation study. *Mol. Ecol.* 14 2611–2620. 10.1111/j.1365-294X.2005.02553.x 15969739

[B22] Excoffier L. Lischer H. E. L. (2010). Arlequin suite ver. 3.5: a new series of programs to perform population genetics analyses under Linux and Windows. *Mol. Ecol. Resour.* 10 564–567. 10.1111/j.1755-0998.2010.02847.x 21565059

[B23] Falush D. Stephens M. Pritchard J. K. (2003). Inference of population structure using multilocus genotype data: linked loci and correlated allele frequencies. *Genetics* 164 1567–1587. 12930761 10.1093/genetics/164.4.1567 PMC1462648

[B24] Freshwater D. W. (1988). Relative genome-size differences among populations of *Spartina alterniflora* Loisel (Poaceae) along east and Gulf coasts of USA. *J. Exp. Mar. Biol. Ecol.* 120 239–246. 10.1016/0022-0981(88)90004-4

[B25] Gallagher R. V. Beaumont L. J. Hughes L. Leishman M. R. (2010). Evidence for climatic niche and biome shifts between native and novel ranges in plant species introduced to Australia. *J. Ecol.* 98 790–799. 10.1111/j.1365-2745.2010.01677.x

[B26] Gallego-Tevar B. Rubio-Casal A. E. de Cires A. Figueroa E. Grewell B. J. Castillo J. M. (2018). Phenotypic plasticity of polyploid plant species promotes transgressive behavior in their hybrids. *AOB Plants* 10 1–16. 10.1093/aobpla/ply055 30377487 PMC6201833

[B27] Geller J. B. Darling J. A. Carlton J. T. (2010). Genetic perspectives on marine biological invasions. *Annu. Rev. Mar. Sci.* 2 367–393. 10.1146/annurev.marine.010908.163745 21141669

[B28] Gong L. Li J. S. Liu X. Y. Zhao X. J. Deng Z. Z. Zhao C. Y. (2014). Genetic diversity of *Spartina alterniflora* in coastal areas of China. *Pratacul. Sci.* 31 1290–1297.

[B29] Goudet J. (1995). FSTAT (Version 1.2): a computer program to calculate F-statistics. *J. Hered.* 86 485–486. 10.1093/oxfordjournals.jhered.a111627

[B30] Guo W. Y. Lambertini C. Pysek P. Meyerson L. A. Brix H. (2018). Living in two worlds: evolutionary mechanisms act differently in the native and introduced ranges of an invasive plant. *Ecol. Evol.* 8 2440–2452. 10.1002/ece3.3869 29531666 PMC5838077

[B31] Hundertmark K. J. Van Daele L. J. (2010). Founder effect and bottleneck signatures in an introduced, insular population of elk. *Conserv. Genet.* 11 139–147. 10.1007/s10592-009-0013-z

[B32] Jakobsson M. Rosenberg N. A. (2007). CLUMPP: a cluster matching and permutation program for dealing with label switching and multimodality in analysis of population structure. *Bioinformatics* 23 1801–1806. 10.1093/bioinformatics/btm233 17485429

[B33] Jenkins D. G. Carey M. Czerniewska J. Fletcher J. Hether T. Jones A. (2010). A meta-analysis of isolation by distance: relic or reference standard for landscape genetics? *Ecography* 33 315–320. 10.1111/j.1600-0587.2010.06285.x

[B34] Kirwan M. L. Guntenspergen G. R. Morris J. T. (2009). Latitudinal trends in *Spartina alterniflora* productivity and the response of coastal marshes to global change. *Glob. Change Boil.* 15 1982–1989. 10.1111/j.1365-2486.2008.01834.x

[B35] Lee C. E. (2002). Evolutionary genetics of invasive species. *Trends Ecol. Evol.* 17 386–391. 10.1016/S0169-5347(02)02554-5

[B36] Liu W. Maung-Douglass K. Strong D. R. Pennings S. C. Zhang Y. (2016). Geographical variation in vegetative growth and sexual reproduction of the invasive *Spartina alterniflora* in China. *J. Ecol.* 104 173–181. 10.1111/1365-2745.12487

[B37] Liu W. W. Strong D. R. Pennings S. C. Zhang Y. H. (2017). Provenance-by-environment interaction of reproductive traits in the invasion of *Spartina alterniflora* in China. *Ecology* 98 1591–1599. 10.1002/ecy.1815 28316076

[B38] Liu W. W. Zhang Y. H. Chen X. C. Maung-douglass K. Strong D. R. Pennings S. C. (2019). Contrasting plant adaptation strategies to latitude in the native and invasive range of *Spartina alterniflora*. *New Phytol.* 226 623–634. 10.1111/nph.16371 31834631

[B39] Liu Y. M. (2018). *Remote Sensing Analysis Of Spartina alterniflora in the Coastal Areas Of China During 1990 to 2015.* Dissertation, University of Chinese Academy of Sciences, Beijing.

[B40] Lu F. Wang X. (2017). General situation of the introduction and genetic diversity of *Spartina alterniflora*. *Shandong Forest. Sci. Tech.* 6 107–112.

[B41] Lu J. Zhang Y. (2013). Spatial distribution of an invasive plant *Spartina alterniflora* and its potential as biofuels in China. *Ecol. Eng.* 52 175–181. 10.1016/j.ecoleng.2012.12.107

[B42] Mantel N. (1967). Detection of disease clustering and a generalized regression approach. *Cancer Res.* 27 209–220. 6018555

[B43] Miller N. Estoup A. Toepfer S. Bourguet D. Lapchin L. Derridj S. (2005). Multiple transatlantic introductions of the western corn rootworm. *Science* 310 992–992. 10.1126/science.1115871 16284172

[B44] Mo Z. Fan H. Liu L. (2010). Investigation on smooth cordgrass (Spartina alterniflora) along Guangxi coastal tidal zone. *Guangxi Sci.* 17 170–174. 10.13656/j.cnki.gxkx.2010.02.009

[B45] Nei M. (1972). Genetic distance between populations. *Am. Nat.* 106:283 10.1086/282771

[B46] Nei M. (1973). Analysis of gene diversity in subdivided populations. *PNAS* 70 3321–3323. 10.1073/pnas.70.12.3321 4519626 PMC427228

[B47] Nei M. Maruyama T. Chakraborty R. (1975). The bottleneck effect and genetic variability in population. *Evolution* 29 1–10. 10.2307/2407137 28563291

[B48] O’Brien D. L. Freshwater D. W. (1999). Genetic diversity within tall form *Spartina alterniflora* loisel along the atlantic and gulf coasts of the United States. *Wetlands* 19 352–358. 10.1007/bf03161766

[B49] Parker I. M. Rodriguez J. Loik M. E. (2003). An evolutionary approach to understanding the biology of invasions: local adaptation and general-purpose genotypes in the weed *Vervascum Thapsus*. *Conserv. Biol.* 17 59–72. 10.1046/j.1523-1739.2003.02019.x

[B50] Paul J. Vachon N. Garroway C. J. Freeland J. R. (2010). Molecular data provide strong evidence of natural hybridization between native and introduced lineages of *Phragmites australis* in North America. *Biol. Invasions* 12 2967–2973. 10.1007/s10530-010-9699-6

[B51] Peakall R. Smouse P. E. (2006). GENALEX 6: genetic analysis in Excel. Population genetic software for teaching and research. *Mol. Ecol. Notes.* 6 288–295. 10.1111/j.1471-8286.2005.01155.x PMC3463245 22820204

[B52] Peakall R. Smouse P. E. (2012). GENALEX 6.5: genetic analysis in Excel. Population genetic software for teaching and research-an update. *Bioinformatics* 28 2537–2539. 10.1093/bioinformatics/bts460 22820204 PMC3463245

[B53] Pritchard J. K. Stephens M. Donnelly P. (2000). Inference of population structure using multilocus genotype data. *Genetics* 155 945–959. 10835412 10.1093/genetics/155.2.945 PMC1461096

[B54] Qiao H. M. Liu W. W. Zhang Y. H. Zhang Y. Y. (2019). Genetic admixture accelerates invasion via provisioning rapid adaptive evolution. *Mol. Ecol.* 28 4012–4027. 10.1111/mec.15192 31339595

[B55] Qin P. Jin M. D. Zhang Z. R. Xie M. (1985). Seed germination experiments of three ecotypes of *Spartina alterniflora*. *J. Nanjing Univ.* 21 237–246.

[B56] Rambaut A. Drummond A. J. Xie D. Baele G. Suchard M. A. (2018). Posterior summarization in bayesian phylogenetics using tracer 1.7. *Syst. Biol.* 67 901–904. 10.1093/sysbio/syy032 29718447 PMC6101584

[B57] Reznick D. N. Losos J. Travis J. (2019). From low to high gear: there has been a paradigm shift in our understanding of evolution. *Ecol. Lett.* 22 233–244. 10.1111/ele.13189 30478871

[B58] Rosenberg N. A. (2004). Distruct: a program for the graphical display of population structure. *Mol. Ecol. Notes* 4 137–138. 10.1046/j.1471-8286.2003.00566.x

[B59] Santos J. Pascual M. Simoes P. Fragata I. Lima M. Kellen B. (2012). From nature to the laboratory: the impact of founder effects on adaptation. *J. Evolution. Biol.* 25 2607–2622. 10.1111/jeb.12008 23110657

[B60] Sattonstall K. (2002). Cryptic invasion by a non-native genotype of the common reed, *Phragmites australis*, into North America. *PNAS* 99 2445–2449. 10.1073/pnas.032477999 11854535 PMC122384

[B61] Sax D. F. Stachowicz J. J. Brown J. H. Bruno J. F. Dawson M. N. Gaines S. D. (2007). Ecological and evolutionary insights from species invasions. *Trends Ecol. Evol.* 22 465–471. 10.1016/j.tree.2007.06.009 17640765

[B62] Selkoe K. A. Toonen R. J. (2006). Microsatellites for ecologists: a practical guide to using and evaluating microsatellite markers. *Ecol. Lett.* 9 615–629. 10.1111/j.1461-0248.2006.00889.x 16643306

[B63] Seneca E. D. (1974). Germination and seedling response of Atlantic and Gulf coasts populations of *Spartina alterniflora*. *Am. J. Bot.* 61 947–956. 10.2307/2441985

[B64] Shafer A. Wolf J. B. (2013). Widespread evidence for incipient ecological speciation: a meta-analysis of isolation-by-ecology. *Ecol. Lett.* 16 940–950. 10.1111/ele.12120 23627762

[B65] Sirkkomaa S. (1983). Calculations on the decrease of genetic variation due to the founder effect. *Hereditas* 99 11–20. 10.1111/j.1601-5223.1983.tb00729.x 6643080

[B66] Slatkin M. (1987). Gene flow and the geographic structure of natural-populations. *Science* 236 787–792. 10.1126/science.3576198 3576198

[B67] Sloop C. M. McGray H. G. Blum M. J. Strong D. R. (2005). Characterization of 24 additional microsatellite loci in *Spartina species* (Poaceae). *Conserv. Genet.* 6 1049–1052. 10.1007/s10592-005-9084-7

[B68] Somers G. F. Grant D. (1981). Influence of seed source upon phenology of flowering of *Spartina alterniflora* Loisel and the likelihood of cross pollination. *Am. J. Bot.* 68 6–9. 10.2307/2442985

[B69] Tamura K. Peterson D. Peterson N. Stecher G. Nei M. Kumar S. (2011). MEGA5: molecular evolutionary genetics analysis using maximum likelihood, evolutionary distance, and maximum parsimony methods. *Mol. Biol. Evol.* 28 2731–2739. 10.1093/molbev/msr121 21546353 PMC3203626

[B70] Travis S. E. Hester M. W. (2005). A space-for-time substitution reveals the long-term decline in genotypic diversity of a widespread salt marsh plant, *Spartina alterniflora*, over a span of 1500 years. *J. Ecol.* 93 417–430. 10.1111/j.0022-0477.2005.00985.x

[B71] van Boheemen L. A. Bou-Assi S. Uesugi A. Hodgins K. A. (2019). Rapid growth and defence evolution following multiple introductions. *Ecol. Evol.* 9 7942–7956. 10.1002/ece3.5275 31380062 PMC6662289

[B72] Vilas A. Perez-Figueroa A. Quesada H. Caballero A. (2015). Allelic diversity for neutral markers retains a higher adaptive potential for quantitative traits than expected heterozygosity. *Mol. Ecol.* 24 4419–4432. 10.1111/mec.13334 26222582

[B73] Wang I. J. (2013). Examining the full effects of landscape heterogeneity on spatial genetic variation: a multiple matrix regression approach for quantifying geographic and ecological isolation. *Evolution* 67 3403–3411. 10.1111/evo.12134 24299396

[B74] Wang I. J. Bradburd G. S. (2014). Isolation by environment. *Mol. Ecol.* 23 5649–5662. 10.1111/mec.12938 25256562

[B75] Wang Q. An S. Q. Ma Z. J. Zhao B. Chen J. K. Li B. (2006). Invasive *Spartina alterniflora*: biology, ecology and management. *Acta Phytotaxon. Sin.* 44 559–588. 22645818

[B76] Wang X. Y. Shen D. W. Jiao J. Xu N. N. Yu S. Zhou X. F. (2012). Genotypic diversity enhances invasive ability of *Spartina alterniflora*. *Mol. Ecol.* 21 2542–2551. 10.1111/j.1365-294X.2012.05531.x 22413802

[B77] Wilson G. A. Rannala B. (2003). Bayesian inference of recent migration rates using multilocus genotypes. *Genetics* 163 1177–1191. 12663554 10.1093/genetics/163.3.1177 PMC1462502

[B78] Xia L. Zhao H. Yang W. An S. Q. (2015). Genetic diversity, ecotype hybrid and mixture of invasive *Spartina alterniflora* Loisel in coastal China. *Clean Soil Air Water* 43 1672–1681. 10.1002/clen.201300882

[B79] Xu C. Y. Tang S. Q. Fatemi M. Gross C. L. Julien M. H. Curtis C. (2015). Population structure and genetic diversity of invasive *Phyla canescens*: implications for the evolutionary potential. *Ecosphere* 6:162 10.1890/ES14-00374.1

[B80] Xu G. W. Zhuo R. Z. (1985). Preliminary studies of introduced *Spartina alterniflora* Loisel in China (I),” in *Research Advances in Spartina: Achievements of Past 22 Years* (Nanjing: Journal of Nanjing University), 212–215.

[B81] Yeh F. C. Yang R. C. Boyle T. B. J. (1999). *POPGENE Version 1.32: Software Microsoft Window-Based Freeware for Population Genetic Analysis.* Edmonton: University of Alberta and Centre for International Forestry Research.

[B82] Zhang Y. Wang Q. Lu M. Jia X. Geng Y. Li B. (2008). Variation and phenotypic plasticity in life history of *Spartina alterniflora* along the east coast of China. *Biodiver. Sci.* 16 462–469.

[B83] Zhao C. Y. Li J. S. Zhao X. J. Liu X. Y. Gong L. Wang R. (2015). *Invasion and Management of Spartina alterniflora Along China’s Coast.* Berlin: Science press.

