# Supplementary material for: Rapid Genetic Divergence of an Invasive Species, Spartina alterniflora, in China
Source: Front Genet. 2020 Apr 24;11:284. doi: 10.3389/fgene.2020.00284 (PMC7193035; doi:10.3389/fgene.2020.00284)
Supplement: Supplementary file 1 [file Data_Sheet_1.docx]

**Table S1 Primer sequences of 35 disomic microsatellite loci in *Spartina alterniflora* that were reported by Blum *et al.* (2004) and Sloop *et al.* (2005)**

| **Locus** | **Primer sequence (5'-3')** | **Core repeat** | **Ta (℃)** |
| --- | --- | --- | --- |
| SPAR**.01** | F:TTACCCTCATCCGAGTCAAAA | **(CT)13** | 58 |
|  | R:GGTGGCGGTGTGGTTCAC |  |  |
| SPAR**.02** | F:GAAGGACGAGTCTCATTTGG | **(CT)14** | 56 |
|  | R:GGCTGCCCCTGTTTCACG |  |  |
| SPAR**.03** | F:CTCAGCTTCCTCCAGAGTGC | **(CTT)6** | 58 |
|  | R:TTGAAGAGACGTGGGAATACG |  |  |
| SPAR**.04** | F:GCCTTCTCGGTCCTTCAG | **(AAG)15** | 65 |
|  | R:TGGGTTGTCGCAGTTATTGG |  |  |
| SPAR**.05** | F:AGGTAACACCGAACGAGTC | **(AG)18** | 58 |
|  | R:CCTACGACATCACCGATA |  |  |
| SPAR**.06** | F:CGGTTGTTTTTGATTGTC | **(CT)18** | 53 |
|  | R:GGTTCTTGGGGAGTTGATTTC |  |  |
| SPAR**.07** | F:TTTCATTTCTGCCGCTTTTAC | **(AG)11** | 54 |
|  | R:GTCGCCCCCTAATCTTTCTC |  |  |
| SPAR.**08** | F:CTAAGGTCCCAAACGACGAC | **(AG)14** | 58 |
|  | R:GCGACGAGCGAGGATTTAC |  |  |
| SPAR**.09** | F:GTGGCCTAGCCTATCGACCT | **(CT)12** | 58 |
|  | R:TGAATGGAAAGGGGAAATGA |  |  |
| SPAR**.10** | F:CGCAAAACGAAACCTTGTTC | **(CT)12** | 55 |
|  | R:AGGCTGCTGGACTGACATCT |  |  |
| SPAR.**11** | F:ATTGTCTCCCTCCCTCTTCC | **(CT)12** | 54 |
|  | R:TCATTTCATCGCACTCACG |  |  |
| SPAR**.13** | F:CCGTGTACTCTAGCCTCTTG | **(CGC)2GCG(CGC)1** | 53 |
|  | R:AGTCGAGTTGCTGTTCAGAT |  |  |
| SPAR**.14** | F:CATGTGGTATCTCCCCATC | **(CTC)3(CT)7** | 51 |
|  | R:GTGACCATAGTTGGCTCTTG |  |  |
| SPAR.**15** | F:ATTTGCTGCTTTTGGTAGAC | **(TC)12** | 51 |
|  | R:GTAGAACAATGGAAGAATGC |  |  |
| SPAR.**16** | F:CTTCCTGCTTGAATTGGTAG | **(CT)11AT(CT)3** | 55 |
|  | R:ACATCGGTGGCAGTAGTAAC |  |  |
| SPAR.**17** | F:TACTTTGGTGTTTGCTTTATC | **(AAG)8** | 60 |
|  | R:GAGTTAGAGGAGTTATTGCTG |  |  |
| SPAR**.18** | F:AACTTCTTGTTCTGGGATTG | **(CTT)8** | 64 |
|  | R:TAGGGAGATAGGACTGGACTG |  |  |
| SPAR**.19** | F:CTAATTCCTCACCCTACGC | **(CT)13** | 51 |
|  | R:CTACGAGACTTCCTCACTGC |  |  |
| SPAR**.20** | F:ACCGTGCCTCAGCTACTG | **(GA)10** | 52 |
|  | R:GGTGTTTCCTCGCATAGATC |  |  |
| SPAR**.21** | F:TGATGCTGTTTCTACCACCTTTAC | **(GA)16** | 53 |
|  | R:CCTCGTCCTCCGTTTTTG |  |  |
| SPAR**.22** | F:ACTGGTCGGTATGGATGC | **(CT)11** | 60 |
|  | R:ATGAGGTCGGTCGTTGTAGC |  |  |
| SPAR**.23** | F:GGGAAGTGAAATCTGGTTGC | **(CT)18** | 55 |
|  | R:GCTTGCTTGTCTCAGTCC |  |  |
| SPAR**.24** | F:TTACACTTGACCTTCTCATC | **(CTT)8** | 64 |
|  | R:GAAACGACTACAGCAATAAG |  |  |
| SPAR**.25** | F:CGGTAGAGACGGAGTTGTGG | **(CTT)10** | 69 |
|  | R:GCTTGGGAGATGAGACTGGAC |  |  |
| SPAR**.26** | F:TTCAACTGGCGTAGTGATTCC | **(TC)12** | 58 |
|  | R:AACATTTCCGACTGGTAGAGC |  |  |
| SPAR.**27** | F:CATCAAAAGCAAGAGGA | **(GA)23** | 50 |
|  | R:GACACCAACGGAACTG |  |  |
| SPAR**.28** | F:CACCGTTCAATCACAGTT | **(TC)2…(TC)2…(TC)5** | 59 |
|  | R:GGAAGCAGGAGGGGTTGG |  |  |
| SPAR**.29** | F:GAACGGTGCATTCTCGATTT | **(AG)8…(AG)4** | 55 |
|  | R:AGCTTACATGGCGGTGTGAT |  |  |
| SPAR.**30** | F:CTATGAGTAGTTGGCCGTTC | **(AT)3…(GA)4…(GA)3** | 53 |
|  | R:TGTTGTACTTCTTCTGGATGC |  |  |
| SPAR**.31** | F:GATCGGACAACTCTATGGAC | **(CTT)7** | 55 |
|  | R:CCAGAAGAAAGTACACAAAG |  |  |
| SPAR**.32** | F:TGGGAACACTTATCAACAATGG | **(CT)4…(CT)5…(CT)4…(CT)4** | 59 |
|  | R:AGGTGGAGACAACGGAGCAG |  |  |
| SPAR**.33** | F:ACCGTAACAACTGAACTCTG | **(TC)8** | 58 |
|  | R:TAGACGACGACCACTGCTTG |  |  |
| SPAR**.34** | F:TCATCATCGACCGAAAAC | **(CT)12C(CT)2** | 55 |
|  | R:TCACCAGTGTCAAGCAGAG |  |  |
| SPAR.**35** | F:TGGAACCTGTAGTCAGAAGC | **(CTT)10** | 59 |
|  | R:GAGGAAGATGATGAAAGTAACG |  |  |
| SPAR**.36** | F:CTTCTATCCAATGTTCGTAG | **(CTT)10** | 58 |
|  | R:TTTAGGTACTGCTGGGATTC |  |  |

| **Table S2 Recent migration rates estimated using BAYESASS across eighteen populations of *Spartina alterniflora*. (A) is the recent migration rate of Chinese populations; (B) is the recent migration rate of American populations.**  **(A)** | | | | | | | | | |
| --- | --- | --- | --- | --- | --- | --- | --- | --- | --- |
| Source→  Target↓ | LN | HB | SD | JS | ZJ | FJ | XG | GD | GX |
| LN | —— | 0.0079 | 0.0080 | 0.0080 | 0.0081 | 0.0080 | 0.0080 | 0.0079 | 0.0080 |
| HB | 0.0082 | —— | 0.0082 | 0.0081 | 0.0120 | 0.0094 | 0.0082 | 0.0083 | 0.0229 |
| SD | 0.0066 | 0.0065 | —— | 0.0065 | 0.0066 | 0.0066 | 0.0064 | 0.0065 | 0.0065 |
| JS | 0.0071 | 0.0071 | 0.0071 | —— | 0.0071 | 0.0071 | 0.0070 | 0.0071 | 0.0072 |
| ZJ | 0.0112 | 0.0086 | 0.0069 | 0.0070 | —— | 0.0070 | 0.0071 | 0.0070 | 0.0131 |
| FJ | 0.0108 | 0.0201 | 0.0109 | 0.0107 | 0.0111 | —— | 0.0107 | 0.0109 | 0.2265 |
| XG | 0.0062 | 0.0064 | 0.0062 | 0.0062 | 0.0802 | 0.0062 | —— | 0.0062 | 0.0193 |
| GD | 0.0074 | 0.0072 | 0.0072 | 0.0072 | 0.0072 | 0.0073 | 0.0072 | —— | 0.0072 |
| GX | 0.0239 | 0.0131 | 0.0087 | 0.0051 | 0.0923 | 0.0056 | 0.0053 | 0.0051 | —— |
| For all values, the direction of migration is from the corresponding population in the first row to the corresponding population in the first column. | | | | | | | | | |

| **(B)** | | | |
| --- | --- | --- | --- |
| Source→  Target↓ | NC | GA | FL |
| NC | —— | 0.0105 | 0.0099 |
| GA | 0.1230 | —— | 0.0445 |
| FL | 0.0041 | 0.0391 | —— |

**Fig. S1 Correlation between *FST* estimated using nuclear SSR loci and geographic distances among the invasive populations in China (A), and the native populations in USA.**

**
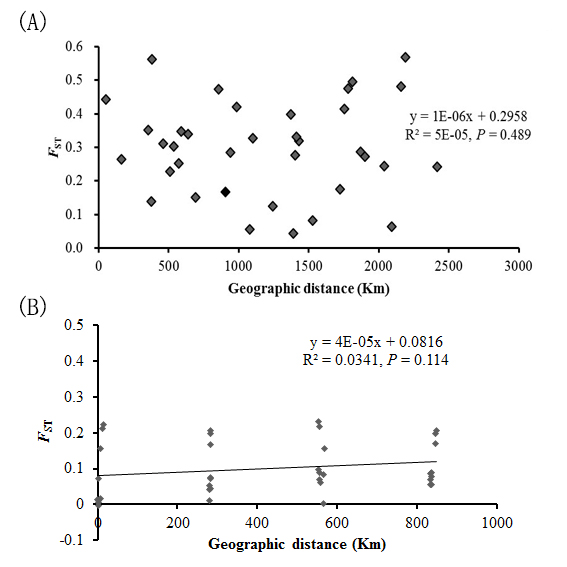
**
